# Attitudes of the German General Population toward Early Diagnosis of Dementia – Results of a Representative Telephone Survey

**DOI:** 10.1371/journal.pone.0050792

**Published:** 2012-11-27

**Authors:** Tobias Luck, Melanie Luppa, Jennifer Sieber, Georg Schomerus, Perla Werner, Hans-Helmut König, Steffi G. Riedel-Heller

**Affiliations:** 1 Institute of Social Medicine, Occupational Health and Public Health, University of Leipzig, Leipzig, Germany; 2 Douglas Mental Health University Institute, McGill University, Montreal, Canada; 3 Department of Psychiatry, Greifswald University, Greifswald, Germany; 4 Helios Hanseklinikum Stralsund, Stralsund, Germany; 5 Department of Gerontology, University of Haifa, Haifa, Israel; 6 Department of Medical Sociology and Health Economics, University Medical Center Hamburg-Eppendorf, Hamburg-Eppendorf, Germany; Marienhospital Herne - University of Bochum, Germany

## Abstract

**Background:**

Early detection of dementia has clearly improved. Even though none of the currently available treatments for the most common form of dementia, Alzheimer’s dementia, promises a cure, early diagnosis provides several benefits for patients, caregivers, and health care systems. This study aimed to describe attitudes toward early diagnosis of dementia in the German general population.

**Methods:**

A representative telephone survey of the German population aged 18+ years (n = 1,002) was conducted in 2011.

**Results:**

The majority of respondents (69%) would be willing to be examined for early diagnosis of dementia. Almost two thirds reported that they would prefer their general practitioner (GP) as the first source of professional help. More than half of the respondents (55%) stated their belief that dementia could be prevented. Respondents mostly indicated psychosocial prevention options.

**Conclusions:**

Our findings suggest that the general population in Germany is very open to early diagnosis of dementia; however, this seems connected with large expectations on the effectiveness of prevention options. Dementia awareness campaigns may be employed to carefully inform the public about the prevention options currently available and their efficacy. To exploit GPs’ potential as a gatekeeper for early detection of dementia, their ability to identify patients with antecedent and mild stages of the disease must be improved.

## Introduction

Dementia represents one of the most significant public health concerns worldwide. Ferri et al. [1] estimate 4.6 million new dementia cases every year and an increase in the number of people affected from 24.3 million in the year 2001 to 81.1 million by 2040. The care of people with dementia already causes enormous costs [2], [3]. Indeed, the societal cost of dementia worldwide was estimated to be $422 billion in 2009, an increase of 34% compared to 2005 [3].

Early prodromal detection of dementia has clearly improved with the development of more sensitive neuropsychological, imaging and/or biochemical markers (e.g., [4]–[6]). The possibility of early diagnosis of dementia, however, raises several ethical questions: First and foremost, there are no curative treatments currently available for the most common form of dementia, Alzheimer’s dementia (AD) [7]. Moreover, available diagnostic methods are still associated with a substantial risk of disease misclassification leading to serious adverse consequences. A test result indicating dementia may be associated with stigmatization, may bring negative feelings like agony or despair for the people diagnosed, or may raise legal questions related to a positive test result like the right to hold a driver’s license, legal capacity or contracting capability [8].

On the other hand, the numerous benefits of an earlier diagnosis of dementia are obvious. Patients and their families can maximize autonomy over decision-making, thus enabling them to plan more effectively for the future (e.g., to discuss advance directives, care arrangements). Early diagnosis also provides them with prompt access to health care services, delaying the time to nursing home admission, and/or optimizing current medical management [9]. With regard to the latter, symptomatic treatments like NMDA receptor antagonists (e.g., Memantine) might be able to stabilize cognition or to delay cognitive decline at least temporarily. According to model-calculations based on the prevalence of AD in the US, broad-based provision of treatments delaying disease onset even modestly could have a major public health impact at the population level [10].

Regarding these potential benefits and disadvantages, knowledge on attitudes toward early diagnosis of dementia in the general population would be of utmost importance for the successful implementation of any future dementia-related health care programs. To date, however, available data on attitudes toward early detection of dementia have been mainly gathered from studies with convenience, clinical (e.g., primary care patients), or selected samples (e.g., older adults, caregivers or relatives of patients with AD) (e.g., [11]–[17]). Population-based studies are rare and in large part focus on attitudes toward genetic testing (e.g., [18], [19]). This study therefore provides information on attitudes toward early diagnosis of dementia in a representative sample of the German general population aged 18 years or older.

## Methods

### Ethics Statement

All subjects, who participated in the study, gave verbal informed consent. Written consent was not obtained as the study was conducted as a telephone survey. Consent to or refusal of participation was documented electronically by the interviewer and a final sample protocol was provided. To ensure quality of the interviews, interviewers were monitored. The study protocol including the consent procedure was approved by the ethics committee of the University of Leipzig, Germany.

### Data Collection and Assessment Procedures

The telephone survey was carried out among German residents aged 18 years and older (02/2011-04/2011). The survey was conducted as a computer assisted telephone interview by USUMA, a leading market, opinion, and social research institute in Germany. Sampling was based on random digit dialing, drawing from the Association of German Market and Social Research Agency’s sample base including registered and non-registered landline telephone numbers. To ensure sample representativeness to the German population, all regions in Germany were included in the sampling process commensurate to their respective population structures. Data were then weighted by household size, age and gender and region based on population statistics of the Federal Statistical Office of Germany. Weighting by age and gender was based on a two-dimensional distribution of these two factors. Within a randomly selected household, the Kish-Selection-Grid was applied while randomly selecting the person in the household (at least 18 years of age) to be interviewed [20]. This ensured equal probability of participation for each member of the household. All interviewers were trained and supervised by a research team of the Institute of Social Medicine, Occupational Health and Public Health at the University of Leipzig.

Altogether, 25,027 valid telephone numbers were called to identify a sample of 5,897 eligible persons. Among these, 3,003 (50.9%) complete telephone interviews were conducted. The remaining 2,894 eligible persons could not be contacted (n = 551; 9.3%), or telephone interviews were refused (n = 2,241; 38.0%) or not completed (n = 102; 1.7%).

Interviews were based on a standardized questionnaire assessing a broad scale of health-related topics. A section on attitudes toward early diagnosis of dementia was asked in every third conducted interview (n = 1,002) and included the following questions:

- Do you think that dementia could be prevented? If yes, what could prevent dementia? (open question; multiple answers were allowed)
- Do you think that early detection of dementia should be offered? (closed question; response categories: yes/no)
- Would you be willing to be examined for early diagnosis of dementia? (closed question; response categories: no/less likely/undecided/more likely/in all cases)
- What would be your first source of professional help? (closed question; response categories: general practitioner/neurologist/psychiatrist/specialized services like memory clinics/other)

Socio-demographic characteristics of the study sample being interviewed about attitudes toward early diagnosis of dementia are shown in table 1. Mean age of the sample was 50.3 years (standard deviation = 17.9 years); nearly one fourth was aged 65 years and older. 41.3% of the sample completed 12 or more years of education. Most participants lived in a two- or multi-person household (77.8%) and in communities with at least 5,000 residents (82.9%).

**Table 1:** Sociodemographic characteristics of the study sample.

Demographic variables	Mean/SD(range)	Categories	n^1^	(%)
Age (years)	50.3/17.9	18–64	755	(75.7)
	(18–92)	65+	242	(24.3)
Gender	–	Female	502	(50.3)
		Male	496	(49.7)
Education	–	<12 years	581	(58.3)
		≥12 years	412	(41.3)
		Not specified	4	(0.4)
Household size	–	Single-person	222	(22.2)
		2 persons	406	(40.7)
		3+ persons	370	(37.1)
Community size	–	0–4,999	170	(17.1)
		5,000–99,999	533	(53.5)
		100,000+	294	(29.5)

1 Deviations from the sample size of n = 1,002 are due to weighting of the data by age, gender, household size and region to ensure representativeness of the sample to the German population.; SD = Standard Deviation.

### Statistical Analysis

The statistical analyses were performed using Predictive Analytics Software, version 20.0. The significance level was set at 0.05 (two-tailed) for all analyses.

We analyzed group differences with *χ^2^*-test. Multivariate logistic regression models were used to estimate the effects of explanatory variables on approval by participants to a general provision of early detection of dementia and willingness to be examined for an early diagnosis, as expressed by odds ratios and 95% confidence intervals (CI). Selection of explanatory variables for the analyses was hypothesis driven.

## Results

### Belief in Prevention Options for Dementia

More than half of the participants (55.0%; 95% CI = 51.9–58.1%) believed that dementia could be prevented (see table 2). These participants most frequently stated brain/memory training (47.4%) and mental activities (33.9%) followed by active life/participation (18.6%) and medication (18.4%) as prevention options.

**Table 2:** Stated prevention options for dementia (n = 549)^1^.

	n^2^	%
Brain/memory training	260	47.4
Mental activity^3^	186	33.9
Active life/participation	102	18.6
Medication	101	18.4
Sport/exercise/physical fitness	76	13.8
Social contacts/avoidance of social isolation	73	13.3
Healthy diet and lifestyle^4^	50	9.1
Early detection/early diagnosis/medical therapy	21	3.8
Scientific research	14	2.6
Group therapy	6	1.1
Pursuing hobbies	6	1.1
Hydration	3	0.5
Other	7	1.3

1 Weighting of the data by age, gender, household size and region to ensure representativeness of the sample to the German population resulted in a sample size of n = 998 participants. Among these participants, 549 (55.0%) stated their belief that dementia could be prevented. Percentages of stated prevention options refer to these 549 participants.; ^2^Multiple answers were allowed.; ^3^learning languages, writing, reading, playing chess, etc.; ^4^less alcohol consumption and smoking.

### Approval to Provision of Early Detection of Dementia

The majority (87.9%; 95% CI = 86.9–89.0%) of the participants stated that they would favour a provision of early detection of dementia. Regarding the results of multivariate logistic regression analysis, approval to provision was significantly associated with male gender and a belief that dementia could be prevented (see table 3).

**Table 3:** Effect of explanatory variables on approval to provision of early detection of dementia – Results of the multivariate logistic regression analysis.

	d.f.	Wald’s χ^2^	p value	OR	95% CI	
Age (65+ vs. 18–64 years)	1	0.345	0.557	0.86	0.52	1.42
**Gender (female vs. male)**	**1**	**4.915**	**0.027**	**0.64**	**0.43**	**0.95**
Education (≥12 vs. <12 years)	1	2.326	0.127	0.73	0.49	1.09
Household size (ref. single-person)						
2 persons	1	0.096	0.756	0.92	0.54	1.56
3 or more persons	1	0.718	0.397	0.78	0.44	1.38
Community size, n (ref. 0–4,999)						
5,000–99,999	1	0.851	0.356	1.29	0.75	2.19
100,000+	1	0.013	0.909	1.03	0.58	1.84
**Belief in prevention options for dementia (yes vs. no)**	**1**	**21.986**	**<0.001**	**2.62**	**1.75**	**3.92**

CI = Confidence Interval; d.f. = degree of freedom; OR = Odds Ratio; ref. = reference category.

### Willingness to be Examined for Early Diagnosis of Dementia

More than two thirds (68.6%; 95% CI = 64.2–72.9%) of the participants indicated that they would be willing to be examined for early diagnosis of dementia (answered ‘more likely’ or ‘in all cases’ to the following question: Would you be willing to be examined for early diagnosis of dementia?) (see table 4).

**Table 4:** Willingness to be examined for early diagnosis of dementia and preferred first source of professional help.

Would you be willing to be examined for early diagnosis of dementia?				What would be your first source of professional help?^2^			
	**n**	**%**			**n**	%	
No	82	8.2		**General Practitioner**	**505**	**62.4**	
Less likely	93	9.3		**Neurologist**	**155**	**19.2**	
**Undecided**	**125**	**12.5**		**Psychiatrist**	**19**	**2.3**	
**More likely**	**292**	**29.3**		**Specialized services^3^**	**119**	**14.7**	
**In all cases**	**392**	**39.3**		**Other^4^**	**3**	**0.4**	
Not specified	13	1.3		**Not specified**	**8**	**1.0**	
**Total**	**997** 1	**100.0**			**809**	**100.0**	

1 Deviation from the sample size of n = 1,002 is due to weighting of the data by age, gender, household size and region to ensure representativeness of the sample to the German population.; ^2^This question was only asked to those who were at least undecided about undergoing an examination for early diagnosis of dementia.; ^3^like memory clinics; ^4^psychologist, toxicologist.

We asked those who were most likely (answers: ‘in all cases’, ‘more likely’, ‘undecided’) to undergo an examination for early diagnosis of dementia about their first choice of professional help in the event of possible dementia. 62.4% stated that they would prefer their GP as their first source of professional help followed by neurologists (19.2%), specialized services like memory clinics (14.7%), and to a much lesser extent, psychiatrists (2.3%) (see table 4).

Multivariate logistic regression analysis revealed that willingness to be examined for early diagnosis of dementia was significantly associated with male gender, lower education, and a belief that dementia could be prevented (see table 5).

**Table 5:** Effect of explanatory variables on willingness to be examined for early diagnosis of dementia – Results of the multivariate logistic regression analysis^1^.

	d.f.	Wald’s χ^2^	p value	OR	95% CI	
Age (65+ vs. 18–64 years)	1	1.603	0.205	0.79	0.56	1.14
**Gender (female vs. male)**	**1**	**7.218**	**0.007**	**0.68**	**0.52**	**0.90**
**Education (≥12 vs. <12 years)**	**1**	**7.976**	**0.005**	**0.67**	**0.50**	**0.88**
Household size (ref. single-person)						
2 persons	1	0.000	0.993	1.00	0.69	1.46
3 or more persons	1	2.821	0.093	0.71	0.47	1.06
Community size, n (ref. 0–4,999)						
5,000–99,999	1	0.075	0.784	0.95	0.65	1.39
100,000+	1	0.183	0.669	1.10	0.72	1.69
**Belief in prevention options for dementia (yes vs. no)**	**1**	**25.572**	**<0.001**	**2.04**	**1.55**	**2.69**

1 The study participants who answered ‘more likely’ or ‘in all cases’ to the question “Would you be willing to be examined for early diagnosis of dementia?” were compared to the participants who answered ‘less likely’ or ‘no’ or who were undecided.; CI = Confidence Interval; d.f. = degree of freedom; OR = Odds Ratio; ref. = reference category.

## Discussion

In a representative sample of the general adult population in Germany, we sought to identify attitudes toward early diagnosis of dementia.

### Approval to Provision of Early Detection of Dementia and Willingness to be Examined for Early Diagnosis of Dementia

We found that the vast majority of the sample (87.9%) would favour a provision of early detection of dementia. Moreover, more than two thirds (68.6%) of the participants indicated that they would be willing to be examined for early diagnosis of dementia.

To date, little is known on the acceptance of early detection of dementia in the general population. Available data from an internet-based questionnaire study conducted in five European countries (France, Germany, Italy, Spain and the United Kingdom) showed that 81% of the respondents from the general public would favour a provision of a routine screening for AD at age 65 [11]. Results were consistent across the five countries. Another study addressed the acceptability of dementia screening in a convenience sample of elderly residents (50+ years) in two retirement communities in the United States (US) [13]. They found that 49% of participants would agree to be routinely screened for memory problems.

A much higher acceptance rate was reported for primary care patients in the US; 81% of patients aged 50 years or older indicated that they would want to be screened to determine whether they are developing dementia [15]. Furthermore, the acceptance rate in the latter study increased to 86% after exposure to the possible risks and benefits of a screening. The authors therefore suggested that primary care patients do want to be evaluated for dementia. Our results indicate that this might be also true for the German general population.

The German population, in general, appears very open to early diagnosis of dementia. This is in accordance with Illes et al.'s findings showing that a substantial proportion of the German population would favour genetic testing for AD (57%) [18]. Moreover, most Germans would not only prefer to be screened for AD, they would also like to be informed in the event of a positive diagnosis (>90%) [21]. It seems very likely, however, that other populations are also very open to early diagnosis of dementia taking into account attitude consistency toward the provision of a routine screening for AD at age 65 years in five European countries mentioned above [11].

Approval by participants to provision of early detection of dementia and their willingness to undergo an examination for an early diagnosis were significantly associated with male gender and belief in prevention options for dementia. Willingness to be examined for early diagnosis of dementia was also associated with lower education.

An impact of male gender, low education, and belief in prevention options for dementia on the acceptance of an early detection examination can be explained within the framework of the *Health Belief Model* (HBM). The HBM postulates that the likelihood of a person engaging in a healthy behavior (in our study: *participation in an early diagnosis examination for dementia*) is determined by an individual’s perception of their susceptibility to a particular condition (*dementia*), severity of that condition, barriers and benefits to engaging in the healthy behavior, and self-efficacy to successfully adopt the desired behavior [22], [23]. We assume that the three factors identified in this study, lower education, male gender, and belief in prevention options for dementia, are specifically linked to perceived benefits of an early diagnosis examination.

Being less educated is likely a proxy for lower knowledge about dementia. We suggest that lower knowledge particularly about the restricted treatment options for dementia should involve higher expectations on (i.e. more perceived benefits of) an early diagnosis examination for dementia and thus a higher likelihood of taking part in such an examination.

A similar line of argument may be used to explain the higher willingness to be examined for early diagnosis of dementia in male participants. Studies have shown that caregivers (professional or family) have more knowledge about AD than non-caregivers [24], [25]. The vast majority of professional and familial caregivers in Germany, however, are women [26], [27]. It is thus very likely that male participants in our study compared to female ones were less exposed to dementia in their line of work or family, and thus also less knowledgeable about dementia and its restricted treatment options. As mentioned above, the lack of knowledge might have contributed to higher expectations of (i.e. more perceived benefits of) an early diagnosis examination for dementia and higher willingness in taking part in such a program.

Finally, we suggest that people who believe in prevention options for dementia should also perceive more benefits of an early diagnosis examination. First and foremost, an early diagnosis of dementia would permit the implementation of suggested (secondary) prevention options. Moreover, our findings are also in accordance with those of Werner [28] showing an association between beliefs about treatments for AD and help-seeking from professional sources in a lay persons sample of Jewish Israeli adults.

### Belief in Prevention Options for Dementia

Overall, more than half of the participants asserted their belief that dementia could be prevented. Interestingly, the most frequently suggested prevention options for dementia were brain/memory training and other mental activities. The perceived importance of these mental activities is in line with findings of others. For example, Schwalen et al. [21] reported that 47% of a representative German sample of 1,245 individuals aged between 14 and 99 years stated that “brain-jogging” might help against AD. Also, in a randomly selected sample of 2,000 community-dwelling Australians aged 18 years or older, 38.8% of participants believed that mental exercise could reduce the risk of developing some forms of dementia [29].

Even though that there is some evidence that mental activity can delay dementia onset [30], [31], participants in our study might have been overly optimistic about the efficacy of such prevention options. Dementia public awareness campaigns are therefore required to carefully inform the public about the prevention options currently available and their efficacy.

### Preferred Source of Professional Help for Dementia

Almost two thirds of the participants stated that they would prefer their GP as the first source of professional help in the event of possible dementia. Specialists were preferred to a lesser extent. These findings are in line with those of a multinational survey on caregivers of patients with AD showing GPs as the first source of contact in 74% of the patients [32].

On one hand, GPs have a decisive role (“gatekeeper”) in the early detection of dementia: They have regular and long-term contact with the vast majority of the elderly population in Germany (>90% [33]) and have the opportunity to identify the first subtle changes and symptoms at the beginning of a dementia process. In addition, utilization of a specialist usually also involves an antecedent referral from a GP.

On the other hand, a positive GP judgment alone seems insufficient for early detection of dementia, as GPs have considerable difficulty in identifying incident dementia and, in particular, in recognizing pre-dementia cognitive deficits and milder forms of the disease [34]–[36]. Moreover, several factors have been identified that may hamper correct recognition and adequate care of dementia patients in primary care settings including time and cost pressure, poor knowledge and low self-perceived competence. As well, affective components on the part of the GP such as embarrassment in conducting a cognitive examination, or a negative general attitude toward caring for patients with dementia could be detrimental to diagnostic and treatment efficacy [37], [38]. Training programs have therefore been suggested that not only enhance GPs’ knowledge and competence regarding dementia but to also address these affective factors [37], [38]. In addition to GP training, early diagnosis of dementia may be also improved by a stronger accessibility to available specialized services (e.g., memory clinics) as well as enhanced interactions between the different agents of the health system [9].

### Limitations

Our study has some limitations. Firstly, households without landline telephone were automatically excluded from participation in the study. Even though the number of individuals in Germany without landline telephone in the household is low (13%; [39]), generalizability of our results has to be made with caution taking the lack of coverage of such households in the study into account.

Secondly, telephone surveys in general seem to be affected by low response, more so than face-to-face surveys [40]. In the present study, complete telephone interviews could be conducted with only half of the people eligible for the study. Even though we sought to ensure representativeness of the study sample to the German general population by weighting the sample by age, gender, household size, and region, a non-response bias cannot be excluded the more so as no information on characteristics of the non-responders could be gathered. Generalizability of our results might be particularly questionable with regard to the education level of the sample; 41.3% of the participants completed 12 or more years of education and thus a considerable higher proportion than in the general population (25.8% [41]). Given that willingness to be examined for early diagnosis of dementia was significantly associated with lower education, the “real” acceptance rate for early detection of dementia in the German general population even might be higher than the rate observed in our study.

Finally, information could only be gathered on a limited number of characteristics of the participants in our study. We are nevertheless aware that participants’ approval to provision of early detection of dementia and their willingness to undergo an examination for an early diagnosis likely depends on further factors (e.g., having a first degree relative).

### Conclusions

Overall, our findings indicate that the German general population at large is very open to an early diagnosis of dementia. This information may be important for the conception, development and implementation of any future dementia-related health care programs.

A positive attitude toward early diagnosis of dementia was strongly associated with large expectations on the availability of prevention options. Dementia awareness campaigns may be employed to carefully inform the public about the currently available options and their efficacy.

Most of the participants would prefer to consult their GPs as the first source of professional help in the likelihood of possible dementia. To meet gatekeeper requirements for early detection of dementia, the ability of GP’s to identify patients with antecedent and mild stages of the disease, however, must be improved.
